# Robotic Colorectal Surgery: A Systematic Review

**DOI:** 10.5402/2012/293894

**Published:** 2012-05-13

**Authors:** Sami AlAsari, Byung Soh Min

**Affiliations:** Department of Surgery, Yonsei University College of Medicine, 50 Yonsei-ro, Seodaemun-gu, Seoul 120-752, Republic of Korea

## Abstract

*Aim*. Robotic colorectal surgery may be a way to overcome the limitations of laparoscopic surgery. It is an emerging field; so, we aim in this paper to provide a comprehensive and data analysis of the available literature on the use of robotic technology in colorectal surgery. *Method*. A comprehensive systematic search of electronic databases was completed for the period from 2000 to 2011. Studies reporting outcomes of robotic colorectal surgery were identified and analyzed. *Results*. 41 studies (21 case series, 2 case controls, 13 comparative studies 1 prospective comparative, 1 randomized trial, 3 retrospective analyses) were reviewed. A total of 1681 patients are included in this paper; all of them use Da Vinci except 2 who use Zeus. Short-term outcome has been evaluated with 0 mortality and191 total major and minor complications. Pathological results were not analyzed in all studies and only 20 out of 41 provide data about the pathological results. *Conclusion*. Robotic surgery is safe and feasible option in colorectal surgery and a promising field; however, further prospective randomized studies are required to better define its role.

## 1. Introduction

Despite the advantages of laparoscopic surgery in the colorectal field, some limitations continue to exist (i.e., two-dimensional visualization, fulcrum effect, restricted degrees of motion of the laparoscopic instruments and amplification of physiological tremors, etc). The recent introduction of robotic surgical system has revolutionized the field of minimally invasive surgery. This system provides high-definition three-dimensional vision, filters physiologic tremor, human wrist-like motion of robotic instruments, and stable camera control, and provides better ergonomics [1]. Robotic technology also eliminates the fatigue associated with conventional laparoscopy. Uhrich et al. have demonstrated that the unnatural positions assumed during laparoscopy contribute to surgeon fatigue and injury [2].

The development of robotic technology in the field of surgery took off in the mid-1980s with telesurgery being the major driving factor. Since that time several robotic devices have been developed, however, the da Vinci system is considered to be the first system approved by the FDA in 2000, and now it is the only available robotic surgical system worldwide.

Since the first report from Weber and colleagues who performed a right hemicolectomy and sigmoid colectomy for benign disease in 2002, more and more surgeons have been becoming interested in robotic surgery and the number of literatures regarding robotic colorectal surgery has been markedly increasing. The study of robotic colorectal resection is probably the most heightened as an alternative treatment option for colorectal cancer. Recent investigations of laparoscopic colorectal resection for the treatment of cancer have revealed superior short-term operative outcomes and noninferior oncologic outcomes compared with open surgery, the classic standard treatment [3–5]. However, in many regions, the actual percentage of laparoscopic surgery performed is still low [6–8]. Steep learning curve of laparoscopic colorectal resection may explain this low penetration rate. Many researches demonstrated that about 50 ~ 70 cases were needed to overcome the learning curve. Even worse, oncologic outcomes as well as short-term operative outcomes may be impaired with inexperienced hands [3, 5–8]. The steep learning curve is of concern especially for rectal cancer, where the quality of total mesorectal excision (TME) influences long-term outcomes [5].

Robotic colorectal surgery, in theory, may have some advantages in this regard. The technological advantages of robotic system may lead to short learning curve and thus may result in better outcomes, which is having been proved in prostate cancer and other gynecologic cancers.

However, for colorectal cancer, the evidence is still weak and the benefits are controversial.

The aim of this study is to perform a comprehensive systematic review and analysis of the current literature on robotics in colon and rectal surgery to assess the safety, feasibility, and outcomes of this emerging new technology especially focusing onto its impact on the treatment of colorectal cancer.

## 2. Literature Search

The electronic database of Medline was reviewed using the PubMed and Google and we examined the bibliographies of all included articles to identify potentially relevant publications. We research all articles from January 2000 to October 2011. Keywords used were: surgical robotics, robotic surgery, robotic colorectal, colon, and rectal. Abstracts from all articles were obtained, and the full texts of studies considered to contain data on the clinical use of robotic surgery were retrieved. 

All articles reporting on robot-assisted resection procedures of the colon and rectum published in the English language were included. The following data were abstracted from each study, if available: type of robotic system, number of patients, type of operation, conversions, operative time, blood loss, intraoperative complications, postoperative complications, number of harvested lymph nodes, resection margins, mortality, and length of hospital stay.

## 3. Materials and Methods

**Types of participants**

The target population consists of adults (>18 years old) male or female undergoing either robotic-assisted colon or rectal surgery.

**Types of interventions**

The interventions under study are robotic-assisted colorectal surgery. This includes robotic-assisted colon or rectal resection.

### 3.1. Results

The total number of studies that have been included in this paper is 41, with total number of 1681 patients being included. All of them they use Da Vinci robotic system except Anvari M et al. and Sebajang et al. who use ZEus robotic system. 

The majority of the robotic colorectal surgery has been done in USA (32%) while Korea is ranked in the second most common country (20%) followed by Italy (15%). 5% of the studies have been done in Canada and Germany, as well as the Netherlands and the rest of the countries did not exceed 2%. (Table 1). 

**Figure 1:**
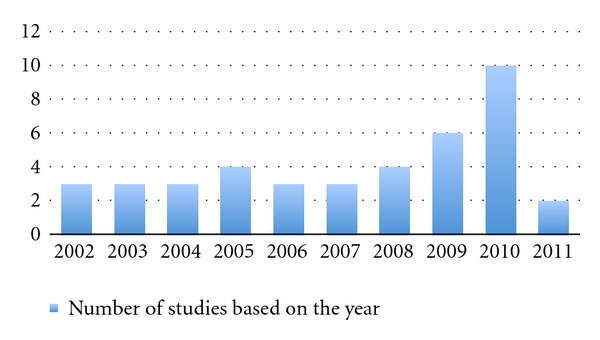
The number of publications regarding robotic colorectal surgery.

The first colorectal study has been published by weber et al. in 2002 and the average number of the study was around 3-4 per year. Recently the study number has been dramatically increased to 6 and 10 in 2009 and 2010, respectively, which means that the robotic system get more popularity and acceptance among most of the colorectal surgeons (Table 1 and Figure 1). 

In the analysis of the type of the studies we found that, 51% of them were case series and 32% comparative non-randomized studies. Only one study has been published by Baik from Korea that was randomized trials (Table 1).

### 3.2. Procedure Type 

Procedure type has been reported clearly in all studies except in 2 of them (Talamini et al. and Bonder et al.) where the type of procedure is not defined. Moreover Hubens et al. he did 7 colectomies in addition to their 6-abdominoperineal resection but they did not define the type of each colectomy. (Table 2). 

The most common colorectal procedure performed by robotic is low anterior resection (41%), right hemicolectomy (20%), sigmoid colectomy (11%), and anterior resection (10%). 

### 3.3. Short-Term Outcome

#### 3.3.1. Operative Time

Procedure duration was one of the outcomes evaluated and the results are summarized in Table 3. 

We noticed that the average operative time were reported in 40 studies. And it ranges from 117.5 to 385.3 min. The variety of types of the procedures included in this study explains this wide gap of operation duration. Also taken into consideration is the fact that even in robotic low anterior resection we had several different procedures from hybrid technique (where usually pelvic dissection (TME) was done with robot, but the rest of the procedure was done laparoscopically) to fully robotic technique (where the most of the procedures except anastomosis were done robotically). 

Most of the comparative studies compare it with laparoscopic technique and they notice that longer time with robotic surgery did not reach statistical significance. However this was statistically significant in 12 comparative studies. Significantly increased operating time was reported by Delaney et al. and Woeste et al. with *P* value of <0.05. Anvari et al., Spinoglio et al., Kim et al., Haas et al., Desouza et al., Park et al., report a *P* value of < 0.001. Rawlings et al. with *P* value of <0.002 Popescu et al., *P* < 0.0002 and Heemskerk et al. *P* < 0.04. 

In the only randomized trial comparing robotic with laparoscopic TME surgery by Baik et al., the operating time was found to be increased by only 13 min in robotic TME (217 min and 204.3 min) but did not reach significance. Of notice here is that they performed hybrid robotic technique where left colon mobilization and vascular handing were done laparoscopically and TME was done robotically. 

One of the key sources of prolonged operation times in robotic surgery is the process involved with docking of the robot. With increasing experience, setup and procedure times were noticed to be reduced after the first few cases. Dealing with different instruments that lack haptic sensation and filtered movement of robotic arms will contribute to increase in the operating time. So, in our opinion accurate comparison with laparoscopic technique needs to be done only after achieving a good learning carve similarity in both techniques. Spinoglio et al. in their study found that the length of time decreased with more experience, and the latter 25 cases showed significantly shorter operative times, largely attributed to setup of the robotic apparatus. 

To further corroborate this finding, Bokhari et al. demonstrate that with increasing number of cases, operative times decreased to achieve statistical significance. 

#### 3.3.2. Length of Stay (Table 3) 

Length of stay is one factor of concern from different angles. Reduction of hospital stay by using a minimal invasive technique in conjunction with fast track modality of treatment can improve patient outcome, recovery, ability to return early to the work, and cost effectiveness. 

This factor has been evaluated in 35 studies and it varies ranging from an average of 3–17.2 days with an overall average of 8 days. 

Only one study has demonstrated a significant reduction in length of stay after robotic surgery (6.9 days in the robotic group compared with 8.7 days in laparoscopic group, *P* < 0.001) [31]. The other authors either did not address this factor for comparison or did not reach statistical significance when they compare it with laparoscopic technique.

#### 3.3.3. Estimated Blood Loss (Table 3)

Intraoperative blood loss has been reported in 21 studies with losses ranging from 16 mL to 400 mL. In most studies, robotic technique was not related to higher rate of intraoperative bleeding. Hellan et al. and Rawlings et al. found blood loss to be reduced in robotic right hemicolectomy with *P* value <0.067 but increased in sigmoid colectomy when compared with laparoscopic resections. Other groups have also reported reduced blood loss with robotic colorectal procedures like Popescu et al. (*P* value < 0.0001) and Desouza et al. (*P* value < 0.052).

#### 3.3.4. Conversion Rate

**Figure 2:**
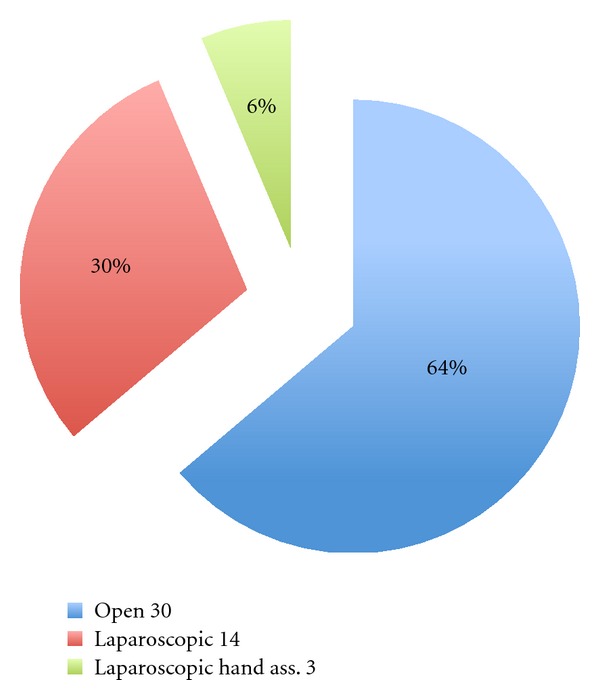
The number and the types of conversion.

The rate of conversion to open or to laparoscopic surgery has been reported in about half of the studies to be 0%. Out of 41 studies, 21 of them show different percentage of conversion rate with different reasons. A total of 30 out of 1681 patients (1.8%) converted to open procedure while 14 patients (0.83%) converted to laparoscopic and 3 patients (0.17%) to laparoscopic hand assisted (Figure 2). Direct comparison should be cautious because of different definitions of conversion among the studies 

Reasons for conversion included obesity with heavy mesentery, inability to identify important vascular structures, vascular injury, adhesions, and narrow pelvis, technical difficulties that included stapler misfiring, inappropriate robotic arm placement, as well as robotic malfunction. Additionally extensive adhesions, locally advanced tumor, retroperitoneal abscess detected intraoperatively due to an infiltrating cancer, colonic ischemia following division of the inferior mesenteric artery, difficulty in identifying the ureter, bowel dilatation, anesthetic difficulties, radical treatment of advanced cancer, and difficult pelvic dissection in a large rectal cancer have been reported as causes of the conversion [11, 13, 17–19, 23–27, 29, 30, 36–38, 42–44, 46, 48, 49]_._

#### 3.3.5. Complications (Table 4)

Surgical complications are wide, variable and not constant. 31 studies show variety and different types of complications. We classify them into different categories: leak, infection, technical, central nervous system, musculoskeletal, cardiovascular, respiratory, urogenital, gastrointestinal, and miscellaneous. 191 complications were reported. Anastomosis leakage was the most common complication observed in 63 (3.7%) cases followed by gastrointestinal (1.4%) and infectious (1.3%) ones. 

Technical complications (usually when we say “technical complications of robotic surgery”, we refer to complications related to malfunction of robotic system; thus hemorrhage does not belong to this category) have been reported in 22 cases over the literature review where the hemorrhage was noticed to be the most common one and only 5 cases instrumental failure while the rest of complications in this category include shoulder torso slide off the table, transverse colon injury, mucosal perforation, and twisting mesentery. 

Other complications based on each category and number of patients in each one include: central nervous system (CNS) with peripheral neuropathy (3) [43], confusion (1) [42], and brain stroke (2) [42, 48]; Musculoskeletal system with Back pain (3) [28, 31] and hip paresthesia (2) [27, 29]; cardiovascular (CVS) with only 2 patients reported by Hella et al. Respiratory complications with atelectasis (2) [13, 48], pneumonia (4) [35, 43, 49], and plural effusion (1) [42] have been reported. Urinary disorder (10) [26, 27, 29, 30, 38], scrotal swelling (1) [31], and retrograde ejaculation (1) [43] were reported under urogenital system. Under-gastrointestinal system complications that have been reported are stoma stenosis (1) [39], ileus (14) [9, 12, 19, 22, 31, 42, 43, 45, 49], GI bleeding (3) [28, 43]. Miscellaneous complications include 3 incisional hernias [30, 43, 48], 1 abdominal wall hematoma [29], 2 deep venous thrombosis [43, 45] and 1 phlebitis [48]. 

Reoperation has been reported with different reasons, 8 cases, reoperated or bowel obstruction [41, 43, 44], 7 cases with leak [15, 26, 27, 48], 1 case for peritonitis [39], and 2 cases for bowel injury [27]. 

### 3.4. Pathological Results after Colorectal Cancer Surgery

Table 5 summarizes the pathological results of all studies including oncologic surgery. The average number of retrieved lymph nodes was adequate for accurate staging ranging from 11.3 to 22.03. Circumferential resection margin is of particular interest because a recent report from a multi-institutional study comparing results from laparoscopic colorectal cancer surgery versus open surgery showed a concern of higher positive rate than open surgery which was even worse when laparoscopic surgery was done in inexperienced hands [5]. CRM has been evaluated in 779 patients (15 studies); out of them 8 patients (1.03%) show positive circumferential resection margin. 

The quality or grade of (Total Mesorectal Excision) TME was evaluated only in 4 studies [26, 31, 34, 37]. Baik et al. report 4 cases of incomplete TME, 6 cases also with incomplete TME reported by Luka et al., and another 6 cases by Baek et al. Few long-term oncologic results after robotic surgery are available at this point. Only 4 studies reported recurrence after surgery [31, 37, 43, 44], which showed acceptable range of recurrence rates. Baek et al. reported their long-term oncologic outcomes after robotic TME for rectal cancer. They reported, after 20.2 months of mean follow-up period, 3-year overall and disease-free survival rates of 96.2% and 73.7%, respectively. Of 6 patients who developed recurrence in their review, 2 had simultaneous local and distant recurrence and the rest 4 had only distant recurrence. Total number of recurrence was reported by 4 studies and it was seen on 14 cases among which local recurrence was observed in 8 of them.

### 3.5. Technical Aspect 

In regard to right colectomy, one of the most debated issues is probably intracorporeal anastomosis. Intracorporeal anastomosis during minimally invasive right colectomy has some potential benefits including smaller laparotomy for specimen extraction, saving mobilization of transverse colon, and prevention of possible disorientation of mesentery resulting from extracorporeal anastomosis. However this technique has not gained popularity because of its technical difficulty. 

The current robotic system, allowing us to make suture and tie-knot, may play an important role of spreading intracorporeal anastomosis technique. Some authors demonstrated technical feasibility of intracorporeal robot-hand-sawn anastomosis already. It seems to be obvious that robotic system enables us to do intracorporeal anastomosis with less effort. One of the possible drawbacks to this is it that to prevent possible spillage of bowel contents within peritoneal cavity during the anastomosis, bowel cleansing may be necessary preoperatively, which is controversial in modern practice. So we need to consider about the true benefit of the technique, especially for the cancer patients before we move on to the new technique. 

As to robotic proctectomy or low anterior resection, there, some technical issues such as hybrid versus totally robotic technique, setting up of robotic system, and splenic flexure mobilization. 

“Hybrid” means that parts of the whole procedures during the robotic surgery are done laparoscopically and the rest are done using robotic system. Usually robotic parts include pelvic dissection or TME and laparoscopic parts include left colon mobilization from splenic flexure to distal sigmoid colon. Vascular handling varies. The potential advantages of hybrid approach are the following: (1) The learning curve is almost flat for experts in standard laparoscopic surgery. (2) The technique is usually reported to be faster than totally robotic technique especially during the initial experience, and according to some authors, the operation duration is almost the same as laparoscopic surgery. (3) No additional docking or movement of robotic cart is necessary after initial docking, which is necessary for the most of totally robotic techniques. However, those advantages of hybrid approach may be valid only if the surgeon is already an expert in standard laparoscopic technique. Thus it is questionable whether these advantages will be the same for inexperienced surgeons or not. 

There are several available “totally robotic” techniques, which can be categorized into 3 types according to redocking of robotic arms and reorientation of robotic cart (or patient table) during the procedure. The first one is single docking method suggested by Hellan et al. The technique involves literally single docking for the entire procedure from colon mobilization to pelvic dissection. Convenience of preparation is the greatest advantage of this technique, whereas that this technique cannot be uniformly applied to all patients as mentioned in their study is major draw back. Recently introduced “flip arm technique” is a modified version of this technique [50]. 

The second one involves 2 dockings, but in fixed position of robotic cart. This technique is probably the most popular “totally robotic” technique. There are a few subtypes, among which the most well known are one from Choi et al. [32] and the other from Lee et al. [49]. Those two have similar setting in terms of the position of robotic cart but different trocars of placement. One of the major drawbacks of those approaches is difficulty in splenic flexure mobilization, which is mandatory in Western patients. There is an ethnic difference in the length of sigmoid colon. Eastern Asian patients like Korean and Japanese ones have long sigmoid colon so that mobilization of splenic flexure of left colon is not a routine procedure in most cases, which is a routine in most of western countries where the patients have relatively short sigmoid colon and the incidence of sigmoid diverticulitis is high. The third one (called “dual-docking technique”), which uses both redocking and reorientation during the procedure, is suggested for more facilitated mobilization of splenic flexure, while sacrificing the convenience of preparation [51]. This technique is also recommended especially for initial experience or as a transition from hybrid to either single docking or double docking with fixed position. 

For conclusion, it would be important to understand advantages and limitations of each technique well to make right application under specific circumstances. And for establishment of robotic experience, as initial approach, either hybrid or dual-docking technique is recommendable according to surgeons' prior laparoscopic experience.

### 3.6. Costs

Operative costs were not consistently reported in all studies. Only eight of them have evaluated costs in different ways. 

Delaney et al. reported a nonsignificant increase in total hospital costs from $2946 for laparoscopic colorectal procedures to $3721.5 for robotic procedures. The median range of Direct Cost (US$) (Operating Room + Equipment) was 1178–2227 US$ and (Total Hospital Cost) 2365–5201 US$. 

They report that the equipment costs relating to Robotic-assisted laparoscopic include robotic laparoscopic instruments costing US $1,500 to $2,000 each, which can be used for ten cases, and sterile drapes (US $150–$200 per case), totalling an average of US $350 per case for this study. This excludes the capital cost of da Vinci purchase (approximately US$1.1 million plus annual maintenance, which is approximately $100,000 per year). 

Rawlings et al. noted that robotic procedures resulted in increases in total operating room (OR) costs, OR personnel costs, OR supply costs, and OR time costs compared with conventional laparoscopy, however, the additional expenses did not achieve statistical significance (Table 6). In both studies, the authors acknowledge that small sample sizes are likely to account for this lack of statistical differentiation. 

Baek et al. did report total operating room costs of US $42,454 for robotic case, of which $1,577 was directly related to robotic instruments. Baik et al. reported cost of the robotic system at US $2,000,000 and additional cost of disposable instruments at $2,000. Bodner et al. compared cost of laparoscopy to robotic and found it to be €4500 and €6500, respectively. In addition to this is the cost of the device, €1,000,000 and €100,000 yearly for maintenance. Choi et al. and Desouza et al. both estimated the cost of robotic procedures to be approximately 3-fold greater than conventional laparoscopy. Results of Desouza et al. are summarized in Table 7. 

Heemskerk et al. also study the cost of robotic rectopexy and compare it with conventional laparoscopic one and they found that robotic rectopexy is more expensive than conventional rectopexy, both in salary and robot-associated costs, leading to higher total costs (3,672.84 versus 3,115.55, or $4,910.55 versus $4,165.46) compared with conventional rectopexy (*P* = 0.012). Instruments cost for robotic was €780.00 ($1,042.85) and for conventional €780.00 ($1,042.85) with *P* value 1. And the costs of the use of da Vinci was €889.18 ($1,188.82) while €0.00 0 for conventional with *P* value 0. 

Finally, Kim et al. report that the main limitation of propagation of robotic TME in the treatment of rectal cancer in Korea is the high cost. And they analyzed and compared total payments and total burdens by patients between robotic, laparoscopy, and open surgery for the treatment of rectal cancer. They found that with a total of 30 patients (10 patients in each group) they recovered and were discharged on time without any complications. The means of total hospital costs were 14,080 USD in robotic surgery, 9,120 USD in laparoscopy surgery, and 8,386 USD in open surgery (*P* < 0.01). Total cost burdens by patients were 11,886 USD in robotic surgery, 3,989 USD in laparoscopy surgery and 3,472 USD in open surgery (*P* < 0.01). 

Moreover, the cost of robotic surgery is still variable between the countries and different centers. In some countries some of the costs have been added to the insurance system while in other countries like Saudi Arabia governmental hospital the patients receive treatment with robotic surgery free. However, we need to look for those patient treated with minimal invasive technique who will have early hospital discharge and early return to work and this will have a large impact in the overall cost comparing to those who receive open technique and late return to the work; beside that new development of different robotic machines from different companies in the future also will affect the costwise. 

On the other hand, in our opinion, the robotic system in comparison to the laparoscopic system has no proven patient-specific advantage [30] but it adds a lot to the surgeons and their technique. So, will the cost burden of the robotic system be added on the patient alone? This is still a debatable issue. 

So, the cost is still an ongoing debate, and the outcome of this debate may determine what role robotics plays in the future. 

## 4. Learning Curve

This part of study has not discussed a lot in literature, especially for those who do little cases of robotic surgery. At present, surgeons utilising telemanipulators for colorectal surgery are accomplished laparoscopic surgeons and previous laparoscopic surgical experience has been shown to shorten the learning curve of robotic surgery [52]. However, one of potential benefits of telemanipulators is to facilitate less experienced surgeons to perform minimally invasive surgery. Therefore, it is necessary for robotic training to begin outside the operating theatre with the aid of simulation. Currently, surgeons attend short training courses to learn basic robotic skills using cadaveric and live animal models [53]. Giulianotti et al in their study found that the learning curve at the console is relatively short, even for an inexperienced surgeon. It does not take long to learn how to do perfect knots and suturing and to have full control of robotic movements, but to perform advanced procedures; full training in open and laparoscopic surgery is mandatory [14]. However, Bokhari et al., published a large retrospective study discussing this issue using a cumulative Sum (CUSUM) method on a series of 50 consecutive robotic assisted laparoscopic surgery procedures. They found that the learning curve consisted of three unique phases: phase 1 (the initial 15 cases), phase 2 (the middle 10 cases), and phase 3 (the subsequent cases). Phase 1 represented the initial learning curve, which spanned 15 cases. The phase 2 plateau represented increased competence with the robotic technology. Phase 3 was achieved after 25 cases and represented the mastery phase in which more challenging cases were managed. And they conclude that the three phases identified with CUSUM analysis of surgeon console time represented characteristic stages of the learning curve for robotic colorectal procedures. The data suggest that the learning phase was achieved after 15 to 25 cases [40]. 

We think that the issue of the learning curve needs to be more widely discussed in the upcoming prospective studies including limitations and how to improve the curve among both those surgeons working with less than 20 robotic cases/year in comparing to those working with more than that. In addition to that we need to assess the impact of learning curve on the short- and long-term patient outcome.

## 5. Conclusion

The surgical robotic system is, as mentioned in MIRA-SAGES Consensus statement [54], an enabling technology that enables more and more surgeon to participate advance in minimally invasive surgery with less effort than it used to cost for standard laparoscopic surgery. In colorectal surgery, the robotic surgical system can help us to overcome steep learning curves and allow us more chances of minimally invasive surgery, especially for rectal cancer surgery. However, robotic colorectal surgery has just begun and the current robotic surgical system is in its primitive stage. There are certainly limitations and disadvantages of robotic colorectal surgery at this point such as high cost, limited instrumentation, and limited range of motion that is not fit for multiquadrant surgery like rectal surgery. So it would be our surgeons' role to make one step forward with unbiased decision to find out the true benefit of robotic surgery for the treatment of colorectal disease. However further prospective studies ongoing like COREAN and ROLARR trials were both of them will add further evaluations and show the true benefit of the robotic colorectal surgery.

## Figures and Tables

**Table 1:** 

Reference	Year	Country	Study type	Number
Weber et al. [9]	2002	USA	Case series	2
Hashizume et al. [10]	2002	Japan	Case series	3
Talamini et al. [11]	2002	USA	Case series	18
Vibert et al. [12]	2003	France	Case series	3
Delaney et al. [13]	2003	USA	Comparative	6
Giulianotti et al. [14]	2003	Italy	Case series	16
Hubens et al. [15]	2004	Belgice	Case series	8
Anvari et al. [16]	2004	Canada	Prospective Comparative	10
D'Annibale [17]	2004	Italy	Comparative	53
Braumann et al. [18]	2005	Germany	Case series	5
Woeste et al. [19]	2005	Germany	Comparative	6
Bonder et al. [20]	2005	Austria	Case series	14
Ruurda et al. [21]	2005	Holland	Case series	23
Pigazzi et al. [22]	2006	USA	Comparative	6
Sebajang et al. [23]	2006	Canada	Case series	7
DeNoto et al. [24]	2006	USA	Case series	11
Heemskerk et al. [25]	2007	Netherlands	Comparative	19
Hellan et al. [26]	2007	USA	Case series	37
Rawlings et al. [27]	2007	USA	Comparative	30
Baik et al. [28]	2008	Korea	Randomized trial	18
Spinoglio [28]	2008	Italy	Comparative	50
Huettner et al. [29]	2008	USA	Case series	70
Soravia et al. [30]	2008	Switzerland	Case series	40
Baik et al. [31]	2009	Korea	Comparative	56
Choi et al. [32]	2009	Korea	Case series	50
DeHoog et al. [33]	2009	Netherlands	Case control	20
Luca et al. [34]	2009	Italy	Case series	55
Park [34]	2009	Korea	Case series	45
Ng et al. [35]	2009	Singapore	Case series	8
Tsoraides et al. [36]	2010	USA	Retrospective	102
Baek et al. [37]	2010	Korea	Retrospective	64
Kim and kang [38]	2010	Korea	Comparative	100
Bianchi et al. [39]	2010	Italy	Comparative	56
Bokhari et al. [40]	2010	USA	Case series	50
Pernazza and Morpurgo [41]	2010	Italy	Case series	50
DeSouza et al. [42]	2010	USA	Case control	40
Zimmern et al. [43]	2010	USA	Case series	131
Popescu et al. [44]	2010	Romania	Comparative	122
Park et al. [45]	2010	Korea	Comparative	41
Haas et al. [46]	2011	USA	Comparative	32
Kang and kim [47]	2011	Korea	Retrospective	204

41				1681

**Table 2:** 

Reference	Procedure type	Reference	Procedure type
Weber	RHC (1), SC (1)	Huettner	RHC(38), SC(32)
Hashizume	ICR (1), LHC (1), SC (1)	Soravia	SC(20), RP(3), LAR(2), RHC(1), AR(1), APR(1), R-V nodule(1)
Talamini	—	Baik	LAR(56)
Vibert	SC(1), PRC(1), RHart(1)	Choi	LAR(40), CA(8), APR(2)
Delaney	RHC(2), SC(3), RP(1)	DeHoog	RP(20)
Giulianotti	RHC(5),ICR(2), SC,(1), LAR(6), APR(2)	Luca	APR(7), AR(17), CA(4), LHC (27)
Hubens	Colectomies (7), APR(1)	Park	LAR(42), APR(3)
Anvari	RHC(5), LAR(2), TC(1), LHC(1), SC(1)	Ng	AR(2), LAR(6)
D'Annibale [17]	RHC(10) LHC(17), SC(11) AR(10), APR(1), TC(2), Hart(1) RP(1)	Tsoraides	RHC(59), SC(43)
Brauman	RHC(1), SC(4)	Baek	LAR(34), CA(18), APR(12)
Woeste	SC(4), RP(2)	Kim	LAR(100)
Bonder	—	Bianchi	APR(7), LAR(18)
Ruurda	RP(16), ICR(5), SCS(2)	Bokhari	SC(25), LAR(15), APR(6), RP(4)
Pigazzi	AR(6)	D'Annibale [41]	RHC(50)
sebajang	RHC(3),SC(3), AR(1)	Desouza	RHC(40)
De noto	SC(11)	Zimmern	RHC(42), AR(24), TPC(7), LAR(47), APR(11)
Heemskerk	RP(19)	Popescu	AR(30), APR(8)
Hellan	LAR(22), CA(11), APR(6)	Park	LAR(29), CA(12)
Rawlings	RHC(17) SC(13)	Haas	AR(25), LAR(7)
Baik	AR(18)	KIM	RP(2), SC(3), RHC(12),TC(1), LAR(201)
Spinoglio	RHC(18), LHC(10), AR(19), APR(1), TRC(1), TC(1)		

RHC: right hemicolectomy; ICR: ileocaecal resection; TRC: transverse colectomy; LHC: left hemicolectomy; SC: sigmoid colectomy; AR: anterior resection; LAR: low anterior resection, APR: abdomino-perineal resection of rectum; PRC: proctectomy; Hart: Hartmann's procedure; RHart: reversal of Hartmann's; RP: rectopexy; TC: total colectomy; SCS: sigmoid colostomy; CA: colo-anal anastomosis; R-V: rectovaginal; TPC: total proctocolectomy.

**Table 3:** 

Reference	Operative time	Length of stay	Blood loss	Conversion rate	C/to open	C/to lap	C/to lap assisted
Weber	284 ± 79.2	—	—	0	—	—	—
Hashizume	260 ± 77.6	17.2 ± 10.2	—	—	—	—	—
Talamini	182	—	—	11%	—	—	—
Vibert	380 ± 62.4	8.3 ± 5.0	400 ± 100	—	—	—	—
Delaney	216.5 (170–274)	3 (2–5)	100 (50–350)	1 (16%)	—	1	—
Giulianotti	202.4	—	—	0	—	—	—
Hubens	124 (87–144)	—	—	0	—	—	—
Anvari	155.3 ± 13.62	5.3 ± 0.95	—	0	—	—	—
D'Annibale [17]	240 ± 61	10 ± 4	21 ± 80	6 (11.3%)	—	2	3
Brauman	201 ± 80.5	13.6 ± 4.7	120 ± 77.1	2 (40%)	2	—	—
Woeste	260.9 ± 34.6	—	60 ± 17.3	1 (25%)	1	—	—
Bonder	310	—	50	0	—	—	—
Ruurda	60–175	6 (3–9)	75 (5–200)	0	—	—	—
Pigazzi	264 (192–318)	4.5 (3–11)	104 (50–200)	—	—	—	—
sebajang	—	4(3–11)	—	1 (14.2%)	1	—	—
De noto	196.7 ± 57.1	3.36 ± 0.5	—	9.1	—	—	—
Heemskerk	152	3.5	—	5%	—	—	—
Hellan	285 (180–540)	4 (2–22)	200 (25–6000)	1 (2.7%)	1	—	—
Rawlings	222.1 ± 4.45	5.6 ± 0.57	65.2 ± 35.6	2 (6.6%)	2	—	—
Baik	217.1 ± 51.6	17.2 ± 10.2	—	0	—	—	—
Spinoglio	383.8	7.74	—	4%	1	1	—
Huettner	224.9	3.5	53.9 (15–500)	8 (11.4%)	5	3	—
Soravia	162	9 (3–24)	—	12.50%	2	3	—
Baik	190.1	5.7	—	0	—	—	—
Choi	304.8	9.2	—	0	—	—	—
DeHoog	154	2.6	—	—	—	—	—
Luca	290	7.5 ± 2.8	68 ± 138	0	—	—	—
Park	293.8 ± 79.7	9.8 ± 5.2	—	2.20%	1	—	—
Ng	192.5	5	—	0	—	—	—
Tsoraides	219.6 ± 45.1	3 (2–27)	66.6	8.8%	5	4	—
Baek	270	5	200	9.40%	—	—	—
Kim	385.3 ± 102.6	11.7 ± 6.7	—	2%	2	—	—
Bianchi	240	6.5	—	0	—	—	—
Bokhari	246.1 ± 80.7	3.5 ± 2.3	106.9 ± 58	—	—	—	—
D'Annibale [41]	223.5	7±1.2	20	0	—	—	—
Desouza	158.9	5	50 (10–240)	2.50%	1	—	—
Zimmern	(158.9–324.3)	5.4–6.4	73.2–252.3	2.4–3.4%	4	—	—
Popescu	212 ± 47.23	8.14 ± 4.5	100 ± 50	2 (5.2%)	—	—	—
Park	231.9 (61.4)	9.9 (4.2)	—	0	—	—	—
Haas	230.9 ± 51.4	3.9 ± 2.9	96.9 ± 46.6	5%	2	—	—
KIM	270	10.5	50	—	—	—	—

Total					30	14	3

**Table 4:** 

	Type and number of complications									
Reference	Leak	I/A	Tech.	CNS	MS	CVS	Resp.	U.G.	GIT	Mis.

Weber									1	
Hashizume			2							
Vibert									1	
Delaney							1			
Giulianotti	1									
Hubens	2	1								
Anvari									1	
Woeste									1	
Ruurda			1							
Pigazzi									1	
Hellan	4	2	1			2		1		
Rawlings					1			2		
Baik					1					
Spinoglio	2	1		1			1		1	2
Huettner	1	1	4		1			2		1
Soravia			2					1		1
Baik	1		1		2			1	1	
Choi	4		2							
Luca	7	2								
Park	1	1					1		1	
Ng							1			
Tsoraides	1									
Baek	4	4								
Kim	8	3	1					4		
Bianchi	1	1							1	
D'Annibale [41]			1							
Desouza			2	2			1		4	
Zimmern	2	1	2	3			2	1	10	2
Popescu	1	2	1							
Park	4	2	2						1	1
KIM	19									

31	63	25	22	6	5	2	7	12	24	7

I/A: infection and abscess; Tech: technical; CNS: central nervous system; MS: musculoskeletal; CVS: cardiovascular system; Resp: respiratory system; U.G: urogenital; GIT: gastrointestinal; Mis: miscellaneous.

**Table 5:** Pathological results.

Reference	N. Lymph node	DRM cm	CRM mm	TME (complete)	Recurrence	Metastasis
Vibert	19	Safe	Safe	—	—	—
D'Annibale [17]	17 ± 10	—	—	—	—	—
Bonder	—	Safe	Safe	—	—	—
Pigazzi	14	3.8	—	—	—	—
Hellan	13	2.7	Safe	37/37	—	—
Baik	18.9	4	+4/56*	—	—	—
Spinoglio	22.03	7.3	—	—	—	—
Baik	18.4	4 ± 1.6	Safe	52/56	2	—
DeHoog	—	—	+1/50*	—	—	—
Luca	18.5	3.16	Safe	22/28	—	—
Park	13	3.4	1.29	—	—	—
Ng	15	2	Safe	—	—	—
Baek	14.5	3.4	Safe	32/38	6	6
Bianchi	18	1.5–4.5	Safe	—	—	—
D'Annibale [41]	18.76	—	—	—	—	—
Desouza	17	—	—	—	—	—
Zimmern	11.8–39.8	Safe	Safe	—	2	1
Park	17.3	2.1 ± 1.4	7 ± 3.9	—	—	—
Popescu	11.3	Safe	Safe	—	2	1
Kim	14.7	2.7 ± 1.9	+3/100*	—	—	—

*Number of positive circumferential margin; CRM: circumferential resection margin; N: number; DRM: distal resection margin; TME: total mesorectal excision.

**Table 6:** 

Cost analysis		L	R	*P* value
Right colectomies 15L/17R	Total hospital cost	5,474–16,280	6,105–$28,304	0.430
	Total OR Cost	3,050–5,826	4,435–8,175	<0.000
	OR personnel cost	621–1,982	1,560–2,869	<0.000
	OR supply cost	1,210–2,851	2,317–4,287	<0.000
	OR time cost	588–1,849	1,164–2,391	<0.000

Sigmoid colectomies 12L/13R	Total hospital cost	5,312–47,651	6,569–$52,042	0.735
	Total OR Cost	3,041–9,368	4,579–$9,147	0.068
	OR personnel cost	754–3,327	1,614–$3,223	0.024
	OR supply cost	966–4,645	2,392–4,780	0.003
	OR time cost	760–1,505	979–2,810	0.519

**Table 7:** The result of Desouza study.

	Laparoscopic (*n* = 91)	Robotic (*n* = 30)	*P* value
	median (range) US$	median (range) US$	Mann-Whitney test
Actual total cost	12,361.50 (7796–79440)	15,192.00 (9801–38453)	0.003
Actual direct cost	7449.0 (4244–46221)	9303.00 (5103–23312)	0.004
- Actual variable direct cost	5718.00 (3031–36489)	7333.00 (3707–17714)	0.005
-Actual fixed direct cost	1657.00 (770–9732)	1897.00 (1396–5598)	0.025
Actual indirect cost	5103.5 (3068–33219)	6218.00 (4698–15141)	0.003
